# Nonlinear effects and effect modification at the participant-level in IPD meta-analysis part 1: analysis methods are often substandard

**DOI:** 10.1016/j.jclinepi.2023.04.013

**Published:** 2023-05-04

**Authors:** Nadine Marlin, Peter J. Godolphin, Richard L. Hooper, Richard D. Riley, Ewelina Rogozińska

**Affiliations:** a Methodology Research Unit, Centre for Evaluation and Methods, Wolfson Institute of Population Health, Queen Mary University of London, 58 Turner Street, London E1 2AB, UK; b MRC Clinical Trials Unit at University College London, Institute of Clinical Trials and Methodology, 90 High Holborn, London WC1V 6LJ, UK; c Institute of Applied Health Research, College of Medical and Dental Sciences, University of Birmingham, Birmingham B15 2TT, UK

**Keywords:** Individual participant data meta-analysis, Effect modification, Interaction, Nonlinear, Sample size, Fractional polynomials, Restricted cubic splines, One- and two-stage models

## Abstract

**Objectives:**

To review analysis methods used for linear effect modification (LEM), nonlinear covariate-outcome associations (NL) and nonlinear effect modification (NLEM) at the participant-level in individual participant data meta-analysis (IPDMA).

**Study Design and Setting:**

We searched Medline, Embase, Web of Science, Scopus, PsycINFO and the Cochrane Library to identify IPDMA of randomized controlled trials (PROSPERO CRD42019126768). We investigated if and how IPDMA examined LEM, NL and NLEM, including whether aggregation bias was addressed and if power was considered.

**Results:**

We screened 6,466 records, randomly sampled 207 and identified 100 IPDMA of LEM, NL or NLEM. Power for LEM was calculated a priori in 3 IPDMA. Of 100 IPDMA, 94 analyzed LEM, 4 NLEM and 8 NL. One-stage models were favoured for all three (56%, 100%, 50%, respectively). Two-stage models were used in 15%, 0% and 25% of IPDMA with unclear descriptions in 30%, 0% and 25%, respectively. Only 12% of one-stage LEM and NLEM IPDMA provided sufficient detail to confirm they had addressed aggregation bias.

**Conclusion:**

Investigation of effect modification at the participant-level is common in IPDMA projects, but methods are often open to bias or lack detailed descriptions. Nonlinearity of continuous covariates and power of IPDMA are rarely assessed.

## 1 Introduction

Evidence synthesis is increasingly used to identify characteristics of patients that respond better to an intervention than others, often termed personalised medicine. Compared to a single trial, the larger sample size afforded by meta-analysis usually provides greater power to reliably determine if a treatment is beneficial and whether the effect of treatment is modified by patient-level covariates.

Meta-analysis can be performed on aggregated summary measures or on individual participant data (IPD). The benefits of analyzing on the individual level are well known [1–3]. Issues such as outcome reporting bias, accounting for prognostic factors, and aggregation bias can be alleviated with individual participant data meta-analysis (IPD-MA), and complex relationships including linear effect modification (LEM), nonlinear covariate-outcome associations (NL) and nonlinear effect modification (NLEM) can be assessed more reliably. Terminology varies in the literature [4] (Box 1). Allowing for such complexity during analysis facilitates the identification of patient subgroup effects, if they exist, and can improve the precision of effect sizes. Leijten et al. included NLEM in their IPDMA and showed that contrary to expectation, children with more severe conduct problems benefitted more from the IY parenting program [5].

**What is new?**

**Key findings**

- Examination of subgroup effects and effect modification is common in individual participant data meta-analysis (IPDMA) but with often inadequate methods. Nonlinearity in effect modification of continuous covariates is seldom considered.
- Power requirements for main effects or effect modification are rarely calculated a priori.

**What this adds to what is known?**

- This review provides an overview of what methods are currently used to address nonlinear covariate-outcome associations (NL) and effect modification in 100 IPDMA of randomized controlled trials between 2015 and 2020.

**What is the implication?**

- Analysis of effect modification in IPDMA can be improved by following existing methodological guidance, including separating within-trial and across-trial relationships to remove aggregation bias, by considering nonlinear relationships, and by assessing the potential power of the IPDMA project in advance of IPD collection.
- Lack of details in reporting IPDMA methods could (partly) be addressed by including analysis code or formal model specifications in publications.

In a single trial, methods for analyzing effect modification and nonlinearity are well established. Effect modification is commonly assessed by splitting the data or including interaction terms into the analysis. Common methods for addressing nonlinearity include categorization [6,7], fractional or ordinary polynomials [8,9] and splines, usually restricted cubic [9]. Extending these to meta-analyses is not always straightforward as variation between studies must be incorporated. In recent years, the methodology available to perform such complex analyses has seen considerable development. Therefore, it is essential to understand what methods are currently used and how they are implemented in IPDMA.

In this article, we present findings of the first of two reviews on IPDMAs. In part 1, we review IPDMAs of randomized controlled trials (RCTs) published between 2015 and 2020, with the aims (1) to describe, if and how LEM, NL and/or NLEM are assessed at the individual level; (2) what methods are being used when examining LEM, NL and/or NLEM at the individual level; and (3) whether these methods meet current methodological standards. The second review (part 2) will focus on summarizing available methodology guidance so researchers can identify the most relevant method for their IPDMA [10].

## 2 Methods

This literature review was guided by a prospectively registered protocol (CRD42019126768) and recommendations on the conduct of methodological reviews [11]. Reporting has been according to PRISMA-ScR where possible [12].

### 2.1 Literature review

We conducted a comprehensive literature search without language restrictions in Medline (via PubMed), Embase, Web of Science, Scopus, PsycINFO and the Cochrane Library. We searched for IPDMAs of RCTs and methodology publications on IPDMA, published between 01 January 2015, and 04 November 2020 (Appendix A). This time limit was chosen to build on previous reviews of IPDMA [13,14]. We describe the results of the review of published methodology in part 2 [10]. A case study comparing the methods in an example IPD will be published separately.

### 2.2 Eligibility criteria

Articles reporting IPDMA were eligible if they included RCTs only and the main objective was estimation of an intervention effect. We restricted to IPDMA of RCTs due to their generally higher reporting standards, and to ‘simplify’ additional analysis and modelling issues that would otherwise arise using observational, non-randomized data. We excluded articles if clustering by trial was ignored during analysis, the paper only described network meta-analysis, or where the full text was not accessible.

### 2.3 Screening

After removal of duplicates, one researcher (NM) screened titles and abstracts and grouped the references into (1) potentially eligible IPDMAs, (2) potentially relevant IPDMA methods papers, and (3) nonrelevant papers.

Among the potentially eligible IPDMAs the same researcher (NM) then randomly sampled articles for a full-text review until 100 eligible IPDMAs that considered LEM, NL and/or NLEM had been identified. At least one other researcher (PG or ER) independently confirmed that each of the 100 articles analyzed LEM, NL and/or NLEM at participant-level. The random sampling procedure was conducted as outlined in the study protocol (PROSPERO CRD42019126768) A random sample of 100 was deemed sufficiently large to identify most methods used by researchers while keeping double data extraction feasible within a limited time frame [11].

### 2.4 Data extraction

Data were extracted from all eligible IPDMAs identified during the sampling process, regardless of whether they assessed LEM, NL or NLEM, thereby enabling investigation of the frequency that such complex relationships are assessed.

Data were extracted using a prospectively developed data collection form that was reviewed after the first ten extractions (Appendix B). Initially we planned to double extract 10% of the included IPDMAs; however due to vague and unclear descriptions in the included articles, all articles were independently extracted by at least two members of the review team (NM, ER, PG). Remaining discrepancies were discussed in the group and, if necessary, further assessed by a senior researcher (RR).

We extracted general information on the IPDMAs, the analysis method used by the authors and whether sample size and multiplicity had been considered (Box 2). If necessary, we checked associated documents such as protocols, analysis plans or previous publications on the same dataset.

For each analysis, we extracted information on whether the trials had been analyzed separately and the effect measures combined (“two-stage”) or individual level data from all trials had been analyzed together while accounting for clustering by trial (“one-stage”) [16]. We also extracted whether the true intervention effect was assumed to be the same across trials (“common or fixed effects”) or whether the true trial-specific intervention effects were assumed to follow a normal distribution (“random effects”) [16]. If interaction terms were included in the models, we also attempted to extract whether the model term assessing effect modification was assumed to be common or random.

We made the following data extraction rules with regards to one- or two-stage and common- or random-effects modelling:

(1) A one-stage common effect approach had been used if the methods mentioned “model stratified for trial” [16,17].
(2) We inferred the method used from software packages described or software commands used. For example, Review Manager or Stata commands ‘metan’ or ‘ipd-metan’ would have been used for two-stage approaches [18,19].
(3) Descriptions such “a random effect for study” or “random effects model” were models with random coefficient for treatment. If “random intercept” was mentioned, it was a common effect model.

We were not able to not extract whether effects of other parameters in the model (such as the intercept, covariates, or residual variances) were modelled as common, random, or stratified. This information was generally not reported.

For the effect modification analysis, we also extracted information on whether aggregation bias had been properly addressed by separating within- and across-trial variation (Table 1). In one-stage models this is done by centering the covariate around its study-specific mean or stratifying the parameters outside the interaction by trial [2,21]. In two-stage models of interaction terms aggregation bias is addressed automatically [16,21].

### 2.5 Data synthesis

Data from the included studies were summarized as frequencies or presented in histograms. No quantitative analysis was performed.

## 3 Results

### 3.1 Classification of articles

**Fig. 1:**
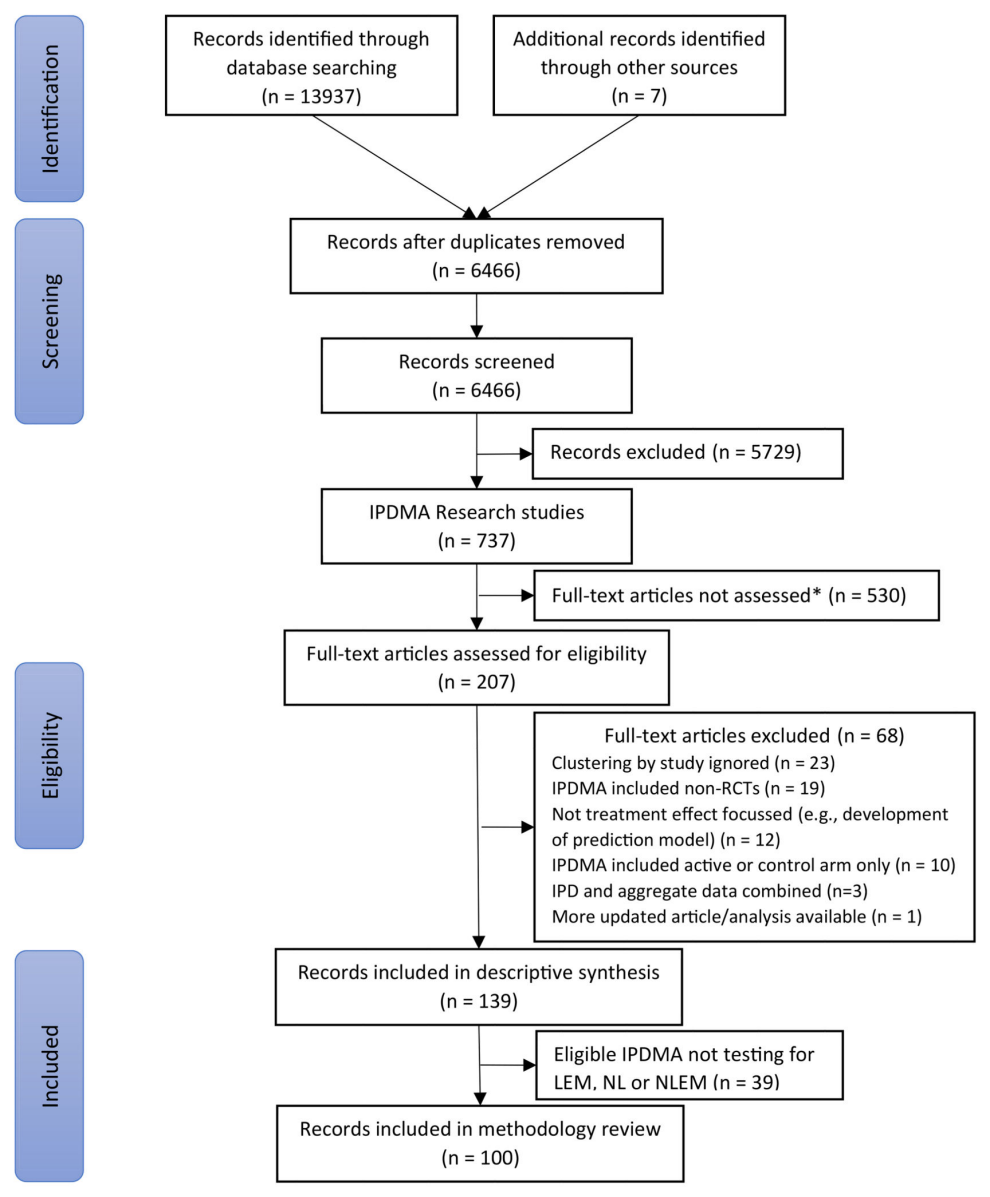
Flow diagram (*This is in the line with the procedure described a priori whereas only a subset of identified IPD research studies was considered in full text. *Abbreviations*: IPDMA, individual participant data meta-analysis; RCT, randomized controlled trial; LEM, linear effect modification; NL, nonlinear covariate-outcome associations; NLEM, nonlinear effect modification).

Database searches identified 6,466 unique records including 737 potentially eligible IPDMAs. Of these, 207 were randomly sampled for full-text review to identify 139 eligible IPDMAs, 100 (70%) of which considered LEM, NL or NLEM and 39 (30%) which did not (Fig. 1).

### 3.2 Characteristics of 100 IPDMAs analyzing LEM, NL or NLEM

#### 3.2.1 Description

Most of the IPDMAs identified came from cancer, cardiovascular or neurology research (Table 2 and Appendix C).

Most IPDMAs identified datasets systematically through literature searches (53/100, 53%), while 14 (14%) did not report how they identified the datasets (Table 2 and Appendix C). Reporting standards were moderate with almost a quarter of article titles not indicating a metaanalysis of IPD and less than a quarter of publications reporting PROSPERO registration (Table 2 and Appendix C).

**Fig. 2:**
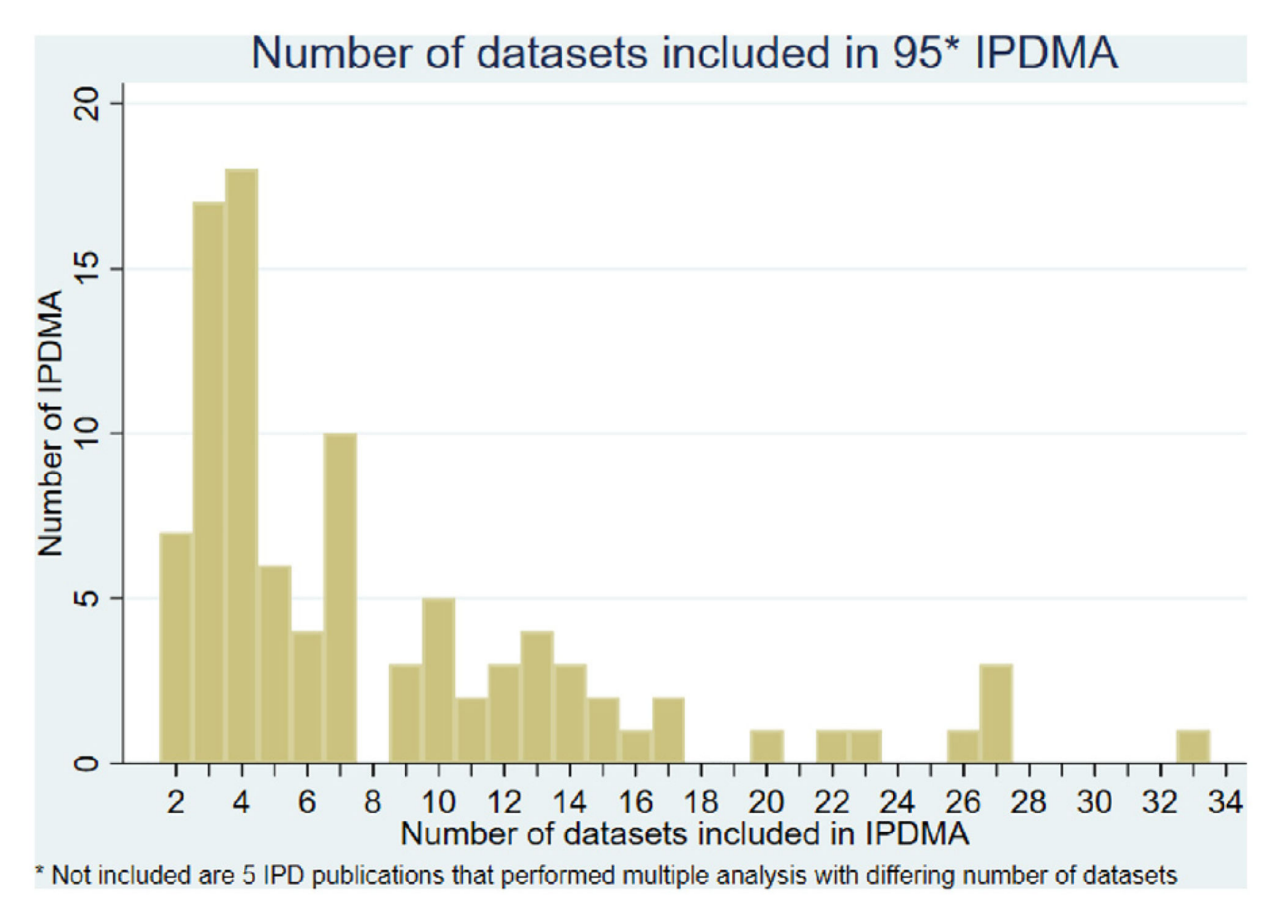
Number of datasets in each individual participant.

**Fig. 3:**
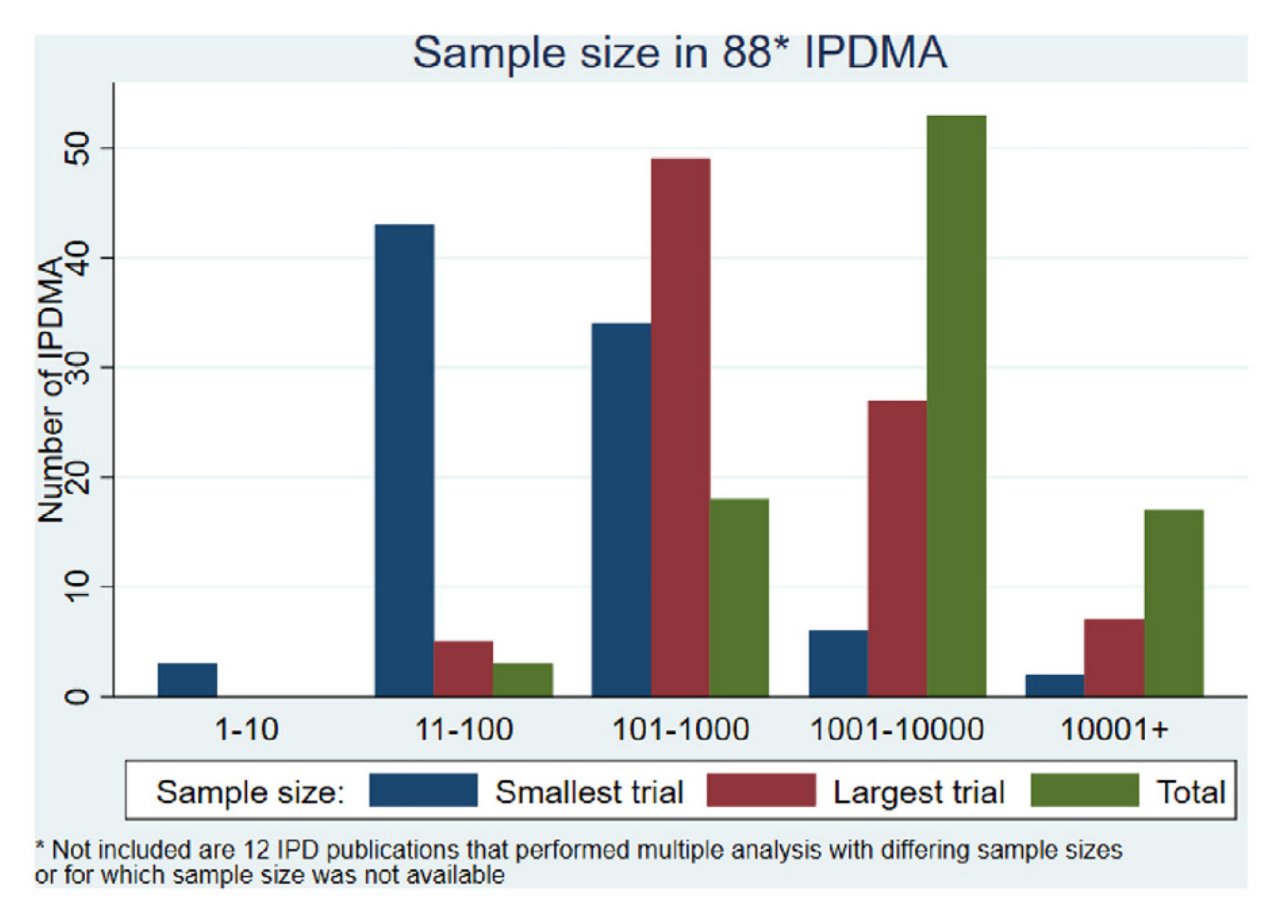
Number of participants in each individual participant dataset meta-analysis.

IPDMAs included between 2 and 34 RCTs (median 5) with individual trials contributing between 7 and 44,567 participants (smallest trial median 91 to largest trial median 709) to their respective IPDMA (Figs. 2 and 3). The median total number of participants included in an IPDMA was 2,186.

A priori power calculation, where power is assessed before IPD collection, was uncommon and only identified in more recent publications from 2017 onwards (Table 2). All five were performed knowing the exact studies and their total sample which ranged from 70 to 4,965. Post hoc power assessments, where the achieved power is calculated based on the effect size estimated during data analysis, were slightly more common (8/100).

Only 3 studies a priori calculated the power required to analyse effect modification at the individual level. Similar trends were seen with adjustments for multiplicity where 94% did not adjust or did not mention the issue.

#### 3.2.2 Comparison to 39 IPD meta-analyses that did not model LEM, NL or NLEM

The median total number of participants included in analysis was about twice as large for IPDMAs investigating LEM, NL and/or NLEM compared to those that do not (2,186 vs. 1,190, Appendix C).

Sample size considerations including multiple testing were more common in IPDMAs analyzing LEM, NL and/ or NLEM compared to those that do not. No other major differences in the key characteristics between those studies were found (Appendix C).

### 3.3 Modelling LEM, NL or NLEM at the individual level

Two IPDMAs evaluated NL only, 88 assessed effect modification only and ten IPDMAs did both. Of those ten, four considered NLEMs, while six had separate models for assessing effect modification and NL (Table 3). Categorization of continuous effect modifiers was common (Table 3). Of 84 studies that modelled continuous effect modifiers (continuous or categorized continuous in Table 3), 59 (70%) only presented analysis of the categorized effect modifier.

Most IPDMAs tested for effect modification by including interaction terms into their one- or two-stage IPDMA model (62/94, Table 4). Of those that had used one-stage approaches only 11% (6/54) reported centering the covariate by its study-specific mean, which is a valid approach for separating within- and across-trial variation [2,21]. 40 one-stage IPDMAs of effect modification did not mention either approach, while 8 reported not to have centered their effect modifiers. Ten reported testing subgroup effects. We only identified one analysis of effect modification on the aggregate study level, which was suitably termed a “daft” approach by Fisher et al. [13]. 24 didn’t report their approach or the description was insufficient.

NLEM was modelled in one study using the multivariable fractional polynomial interaction procedure to identify the best fitting nonlinear form [22]. Three studies prespecified for the interactions to include quadratic terms of the potential effect modifying covariate. All four reported using one-stage models but only one reported dealing with aggregation bias by centering the effect modifier around its study-specific mean. One stated that the covariate had not been centered, while two did not mention the issue at all.

Nonlinear covariate-outcome effects were modelled in eight studies. Two used three-knot splines, one positioned the knots at tertiles of the covariate and one did not report knot locations. Two studies included unspecified polynomial terms of the covariate in their statistical model, one included a quadratic term, and one log-transformed their outcome. The remaining two studies did not present sufficient detail, simply stating they had used nonlinear mixed models or used a variety of nonlinear methods.

## 4 Discussion

### 4.1 Main findings

In this article, we have reviewed the methodology used in IPD research studies to address LEM, NL or NLEM. Investigating such associations with sufficient power can support the reliable identification of patient subgroups that benefit the most from an intervention. Our random sample of IPDMAs showed that analysis of LEM at the individual-level is common in IPDMA of RCTs, with only a few investigating nonlinearity.

#### 4.1.1 Planning the analysis of effect modification - sample size

Many IPDMAs analyse effect modification, whether linear or nonlinear, but very few power for it. Post hoc assessments of power are more common. Zhang and colleagues showed that such assessments do not capture the true power to detect a desired effect. They can be misleading, and are therefore discouraged by many authors [23–25]. The only three studies calculating power for the effect modification analysis a priori came from the same research team and due to lack of detail we could not reproduce the calculation.

Admittedly, control over the sample size in an IPDMA may be limited. However, researchers can assess before onset of the project whether their “promised” data will allow detection of a clinically relevant effect. This may impact decisions on whether it is worth embarking on the time-consuming tasks of obtaining, cleaning, harmonizing, and analyzing the IPD [26]. It may also allow the researchers to focus their efforts on obtaining IPD for a subset of trials if there is clear justification (such as when the IPDMA needs to be completed quickly due to a very urgent clinical need for evidence).

Power calculations, done before IPD meta-analysis, are currently not part of PRISMA-IPD guidelines but can support promising IPDMA of effect modification or deter futile ones. It is also important to note that analyzing large numbers of effect modifiers could increase the risk of spurious findings. Godolphin et al. [15] show that on average six modifying covariates (range 1 to 28) were analyzed for on average two outcomes (range 1 to 16). As far as we are aware, there is currently no guidance how to power an IPDMA if multiple effect modifiers are of interest. We suggest assessing the power for each of a small set of effect modifiers deemed of most interest (primary analyses).

### 4.1.2 IPDMA methodology

Of 207 articles considered in full text we excluded over 10% (23/207) as they simply pooled the individual datasets during analysis without accounting for clustering by trial. This flawed practice can lead to misleading effect estimates and conclusions and should not be used [27].

One-stage models were clearly favoured for analysis of effect modification and/or nonlinearity. Analyzing all data in one step may appear more elegant but requires more care than the traditional two-step approach especially when analyzing effect modification. Within- and across-trial variation need to be separated in a one-stage approach, for example by centering the effect modifier around their study specific mean and then including the interaction terms into the model [2]. Only seven of 58 one-stage analyses of effect modification reported such centering, while nine stated that they had not centered the effect modifier and 42 did not mention either approach so it is reasonable to assume it wasn’t done during analysis. Therefore, almost three-quarters of IPDMA analyzing effect modification using a one-stage approach provided insufficient details to assess their appropriateness. It is important to note that many methodology articles were published during the study period (2015e2020), and therefore awareness of the issues might have been limited especially in the earlier IPDMA studies.

Nonlinearity is rarely considered. Researchers still prefer to categorize continuous effect modifiers rather than analyzing on the continuous scale and allowing for nonlinearity. Admittedly, in some cases this may have been due to how the data were recorded in the original trials or how they were shared. Categorization may be a useful investigatory tool [28], but dangers and pitfalls of categorization as the primary analysis have been extensively shown for both single studies [6,7] and for IPDMAs [29].

#### 4.1.3 Reporting of IPDMA

PRISMA IPD (published in 2015) standardized the reporting of IPDMAs of randomized trials and provided an easy-to-follow guideline and checklist that should ensure high quality reporting of any such study [30]. However, even the reporting of basic information, such as the study type in the title of the IPDMA was missing for a substantial number of articles.

The overall IPD approach is usually extractable (i.e., the use of common-effect or random-effects models or one- or two-stage approach), but modelling details such as the centering of covariates and handling of nuisance parameters (e.g., study-specific intercepts and adjustment factor effects), or for studies that used multiple approaches, what results were produced by each analysis was often not reported or unclear. We strongly support previous suggestions to publish software code or writing out the formal model specification (e.g., regression equation) to improve understanding and reproducibility especially of one-stage models [31,32]. Some may resist due to the extra effort required to review manuscripts [33].

### 4.2 Limitations

This literature review has some limitations. Firstly, our results may be limited as we did not consider all IPD studies published 2015–2020 and instead used random sampling until we identified 100 eligible articles. However, we did perform a comprehensive search across a variety of databases with inclusive search terms, thus identifying a representative sample to randomly select articles from.

Secondly, in our random sample we identified 32 IPD-MA that were conducted by researchers from the same team, used the same dataset or used shared protocols (14 groups). A number of these IPDMAs shared the same analysis strategy, likely reducing the variation in methods identified in this review.

Thirdly, we identified few analyses of nonlinear covariate-outcome relationships. This is likely due to our inclusion criteria and restricting IPD research studies to those focussing on treatment effect estimation. Assessment of nonlinear covariate outcome relationships is more common in prognostic factor studies or development of prediction models.

Finally, due to unclear reporting, we had to make some data extraction rules regarding analysis strategies. These might not necessarily reflect the interpretation of other researchers. However, these rules were agreed within a review team with extensive experience of IPDMA.

## 5 Conclusion

Analysis of complex relationships, in particular effect modification, is common in IPDMAs but rarely done appropriately. Most continuous covariates are categorized and analyzed in a one-stage model that amalgamates within- and across-trial information, potentially introducing aggregation bias at the patient-level. To have a meaningful impact on stratified and precision medicine research, effect modification analyses need to be conducted correctly, sufficiently powered and reported transparently.

## Figures and Tables

**Table 1:** Classification of effect modification approaches

IPDMA approach	Effect modification approach	Within and across trial variation separated
Two-stage	Analysis of interaction terms (subgroup analyses that do not involve modelling an interaction term do not count) [20]	Yes
	Analysis of subgroup effects	No
One-stage	Model including interaction terms between treatment and covariate and centering around study-specific mean or stratification is reported	Yes
	Model including interaction terms between treatment and covariate but no mentioning of centering or stratification	Unclear
	Model including interaction terms between treatment and covariate, where the covariate is centered around its overall mean or stating that it has not been centered	No
Aggregate level	Meta-regression of aggregated patient-level covariate measures	No

**Table 2:** General characteristics of the included IPD research studies (Data are frequency (%) unless stated otherwise)

Variable	Category	Number of IPD meta-analyses or statistic (*N* = 100)
Year published	2015	14 (14%)
	2016	8 (8%)
	2017	17 (17%)
	2018	19 (19%)
	2019	22 (22%)
	2020	20 (20%)
Medical field	Cancer	11 (11%)
	Cardiovascular	29 (29%)
	Mental Health	6 (6%)
	Neurology	14 (14%)
	Women’s Health	7 (7%)
	Other	33 (33%)
Dataset identification	Systematic	53 (53%)
	Nonsystematic	33 (33%)
	Not reported	14 (14%)
Title identifies article as meta-analysis of individual participant data	Yes	76 (76%)
	No	24 (24%)
PROSPERO registration	Yes	23 (23%)
	None reported	77 (77%)
Sample size consideration – Main effect	A priori power calculation	5 (5%)
	Post hoc power assessment	8 (8%)
	None or general discussion of power in IPDMA	87 (87%)
Sample size consideration – Effect modification	Power calculation	3 (3%)
Multiple testing considered	Yes	6 (6%)
	No	21 (21%)
	Unclear or not applicable	1 (1%)
	Not reported	72 (72%)

**Table 3:** Forms of effect modification modelled in each IPDMA

Effect modifiers	Nonlinear effects (*n* = 12)	No nonlinear effects (*n* = 88)	Total
At least one effect modifier analyzed as continuous	8 (4 + 4)^a^	17	25
All continuous effect modifiers were categorized	1	58	59
Only categorical effect modifiers analyzed	1	13	14
None	2	-	2

a 4 studies assessed nonlinear effect modification, 4 studies modelled effect modification and nonlinear terms separately.

**Table 4:** Meta-analysis approach for linear effect modification (LEM), nonlinear covariate-outcome associations (NL) or nonlinear effect modification (NLEM) in 100 IPD research studies (studies can appear multiple times)

LEM, NL or NLEM	IPDMA approach	Analysis of	Within and across trial variation separated	Nr of IPDs
Linear effect modification (*n* = 94)^a^	Two-stage (*n* = 15)	Interaction term(s)^e^	Yes	8
		Subgroup effects	No	6
		log rank tests	No	1
	One-stage (*n* = 54)	Interaction term(s)^e^	Yes^b^	6
		Interaction term(s)^e^	Unclear^c^	40
		Interaction term(s)^e^	No^d^	8
	Unclear	Subgroup effects	No	3
	Aggregate level	Subgroup effects	No	1
	Insufficient detail			24
Nonlinear effect modification (*n* = 4)	One-stage (*n* = 4)	Interaction term(s), quadratic covariate	Yes^b^	1
		Interaction term(s), quadratic covariate	Unclear^c^	1
		Multivariable fractional polynomials interaction (degree 1)	Unclear^c^	1
		Interaction term(s), quadratic covariate	No^d^	1
Nonlinear effects (*n* = 8)	Two-stage	Restricted cubic splines		2
	One-stage (*n* = 4)	Linear and polynomials terms with backward selection		1
		Fractional polynomials		1
		Quadratic term (prespecified)		1
		Log-transformed outcome (based on model performance)		1
	Insufficient detail			2

a 3 studies reported multiple approaches falling into different categories.

b Article reported centering of the effect modifier around the study-specific mean.

c Article did not report on dealing with aggregation bias by centering or stratification.

d Article reported that aggregation bias was not dealt with or dealt with incorrectly.

e Interaction term(s) between a binary intervention indicator and covariate (i.e., effect modifier).

## Box 1 Terminology for individual participant data meta-analysis of complex relationships

Here we present some brief explanations of commonly used terms in the literature.

### LEM, NL, and NLEM

Interaction: The combined effect of two factors is different than their individual effects. During analysis a multiplicative term is included into the model in addition to the individual factors.

Effect modification: It is a type of interaction between a binary intervention indicator and a covariate called the effect modifier. The effect of an intervention differs depending on the level of the modifier characteristic. During analysis an interaction term between the intervention indicator and covariate is included in the model. If the covariate is categorical, the term is also used when the effect is estimated within subsets of data.

Subgroup effect: The effect of the intervention within a defined subset of patients usually defined by categorical characteristics. The term subgroup effect is used for analyses including interaction terms or analyses within subsets of data.

Nonlinearity: Estimates are not consistent across varying levels of patient characteristics, either in an effect modification or covariate-outcome relationship.

### IPDMA approaches and distributional assumptions

Two-stage IPDMA: The effect of interest is analyzed in each trial separately and the estimates combined using meta-analysis techniques.

One-stage IPDMA: Data from all trials are analyzed together while accounting for clustering by trial.

Common/Fixed effects: The true effect is assumed to be the same across trials. Differences seen in individual trial estimates are only due to sampling error.

Random effects: The true effects in each trial are assumed to follow a normal distribution allowing for between study variation.

Effects stratified by trial: The effect in each trial is independent from those in other trials.

## Box 2 Data extraction (Not all data is reported in this article). For further findings see Godolphin et al. [15]

General: Date of extraction, first author, research group/collaboration, year of publication, medical field, number of datasets analyzed, number of participants included/analyzed per dataset, dataset identification, PROSPERO ID.

Main effect analysis: General IPDMA approach (one-/two-stage, common/random/unclear intervention effect); general modelling approach (logistic, survival, etc.)

Outcome: Format of primary outcome, is any outcome a composite.

Power considerations: Sample size calculation (power calculation, post hoc power assessment, general talk of increased power when using IPD compared to single trial only); multiple testing adjustments.

Complex associations at the individual level: Nonlinear effects; effect modification; subgroup analysis without testing.

Details on nonlinear effects analysis: Analysis approach; justification for analysis approach; any NLEM; if so, analysis approach used for NLEM.

Details on effect modification analysis: Format of effect modifier (at least one analyzed on continuous scale, all continuous effect modifiers categorized, categorical effect modifiers only); number of effect modifiers; number of outcomes for effect modification; analysis approach for effect modification; classification of analysis approach using terminology by Fisher et al. [13] (deft, one-stage unclear if within and across information separated out, deluded, daft, unclear); Inclusion of additional covariates besides treatment-covariate interaction.

## Footnotes

**Supplementary data**
Supplementary data to this article can be found online at https://doi.org/10.1016/j.jclinepi.2023.04.013.
